# THE VALUE OF ENGAGEMENT: IMPROVING ORAL HEALTH THROUGH COMMUNITY-BASED INTERVENTIONS

**DOI:** 10.1093/geroni/igae098.1465

**Published:** 2024-12-31

**Authors:** Suchitra Nelson, Shelley Curtan, Farida Ejaz

**Affiliations:** Case Western Reserve University, Cleveland, Ohio, United States; Case Western Reserve University, Cleveland, Ohio, United States; Benjamin Rose Institute on Aging, Cleveland, Ohio, United States

## Abstract

Many older adults accept oral health problems as an inevitable consequence of aging and do not seek dental care. Further, with limited dental insurance options alternative care models are needed. Our project investigated the most effective and efficient means of addressing oral health disparities and access to dental care among community-dwelling older adults. This clinical trial tested the comparative effectiveness of two non-surgical evidence based cavity treatments (Silver Diamine Fluoride vs. Atraumatic Restorative Treatment) in arresting tooth decay for adults 62+. Treatments required no needles or drills and were done using portable equipment in the independent housing sites where the adults lived. The project enrolled and screened 952 older adults, treated 568 with active decay, living in 34 housing sites (17 sites randomized to each treatment intervention). Follow-up visits at 26 and 52 weeks assessed caries arrest and participant report of oral pain and hypersensitivity. Focus groups were conducted to capture qualitative data related to participant’s treatment satisfaction. A stakeholder group comprised of individuals representing payers, policy makers, advocacy organizations, housing administrators, and patients have been active throughout each study phase, offering guidance and assistance with the project. We will outline experiences of participants and perspectives of stakeholders that enabled positive oral health outcomes for older adult participants. Challenges and successes will be shared, along with next steps for dissemination and ideas for health system change to support continuation of treatment based on project clinical outcomes.

